# Investigation of Interspecies Interactions within Marine Micromonosporaceae Using an Improved Co-Culture Approach

**DOI:** 10.3390/md13106082

**Published:** 2015-09-24

**Authors:** Navid Adnani, Emmanuel Vazquez-Rivera, Srikar N. Adibhatla, Gregory A. Ellis, Doug R. Braun, Tim S. Bugni

**Affiliations:** 1 Pharmaceutical Sciences Division, University of Wisconsin-Madison, Madison, WI 53705, USA; E-Mails: nadnani@wisc.edu (N.A.); snadibhatla@wisc.edu (S.N.A.); gaellis@wisc.edu (G.A.E.); drbraun@wisc.edu (D.R.B.); 2 Molecular & Environmental Toxicology Center, University of Wisconsin-Madison, Madison, WI 53705, USA; E-Mail: vazquezriver@wisc.edu

**Keywords:** Co-culture, Micromonosporaceae, marine invertebrate, metabolomics, cryptic biosynthesis, marine bacteria

## Abstract

With respect to bacterial natural products, a significant outcome of the genomic era was that the biosynthetic potential in many microorganisms surpassed the number of compounds isolated under standard laboratory growth conditions, particularly among certain members in the phylum Actinobacteria. Our group, as well as others, investigated interspecies interactions, via co-culture, as a technique to coax bacteria to produce novel natural products. While co-culture provides new opportunities, challenges exist and questions surrounding these methods remain unanswered. In marine bacteria, for example, how prevalent are interspecies interactions and how commonly do interactions result in novel natural products? In an attempt to begin to answer basic questions surrounding co-culture of marine microorganisms, we have tested both antibiotic activity-based and LC/MS-based methods to evaluate Micromonosporaceae secondary metabolite production in co-culture. Overall, our investigation of 65 Micromonosporaceae led to the identification of 12 Micromonosporaceae across three genera that produced unique metabolites in co-culture. Our results suggest that interspecies interactions were prevalent between marine Micromonosporaceae and marine mycolic acid-containing bacteria. Furthermore, our approach highlights a sensitive and rapid method for investigating interspecies interactions in search of novel antibiotics, secondary metabolites, and genes.

## 1. Introduction

Natural products continue to serve as a contributor for novel small molecule therapeutic agents [1]. Genomic evaluation of natural product producing bacteria has highlighted that the biosynthetic potential far surpass the number of natural products typically isolated in the laboratory [2]. The undiscovered chemistry due to “cryptic”, or silent, biosynthetic genes encompass a plethora of untapped chemical scaffolds. One promising approach to activating cryptic clusters has been to leverage bacterial-bacterial interspecies interactions [3]. Over the past decade alone, the number of publications in the field of microorganism co-culture has increased by over five-fold, including several notable examples of either fungal-fungal [4,5,6,7,8,9], fungal-bacterial [10,11,12,13,14], or bacterial-bacterial co-culture [15,16,17,18,19,20,21,22,23]. In addition to co-culture, specific examples of bacterial-bacterial crosstalk have been investigated [3]. As an example, Vetsigian *et al.*, created a high-throughput platform to investigate pairwise interactions among soil-derived *Streptomyces* spp. [24]. Dorrestein and co-workers interrogated interspecies interactions, including competition-mediated antibiotic production between bacteria using imaging mass spectrometry (IMS) [25,26]. Akin to quorum sensing, bacterial interactions could play important roles in modulating small molecule biosynthesis. Therefore, interspecies phenomena such as communication and competition are of significant interest, especially where dense microbial populations are encountered such as marine invertebrates.

Within marine invertebrates (*i.e.*, sponges, ascidians, *etc.*) exist complex bacterial populations and quite likely important interspecies interactions. In marine sponges, for example, up to 50%–60% of their biomass is bacterial cells and can exceed a density of 10^9^ bacterial cells per cm^3^ of tissue [27,28,29]. Chemistry isolated from whole animals has been shown to be produced by symbiotic bacteria associated with the organism [30,31,32]. In ascidians for example, some bacteria known to produce chemistry found in whole animals have a much reduced genome indicating dependence on the host environment [33,34]. Therefore, bacterial interactions within whole animals likely play a role in symbiont homeostasis and chemistry. While culture independent studies are beginning to shed light on the importance of symbionts, we wanted to begin investigating potential interactions among cultivable bacteria via co-culture. Importantly, we wanted to begin addressing many of the challenges with co-culture including detection, isolation, and fermentation scalability. Therefore, we evaluated methods and more broadly the occurrence of interspecies interactions within marine bacteria that altered secondary metabolite production as a route to novel natural products.

**Figure 1:**
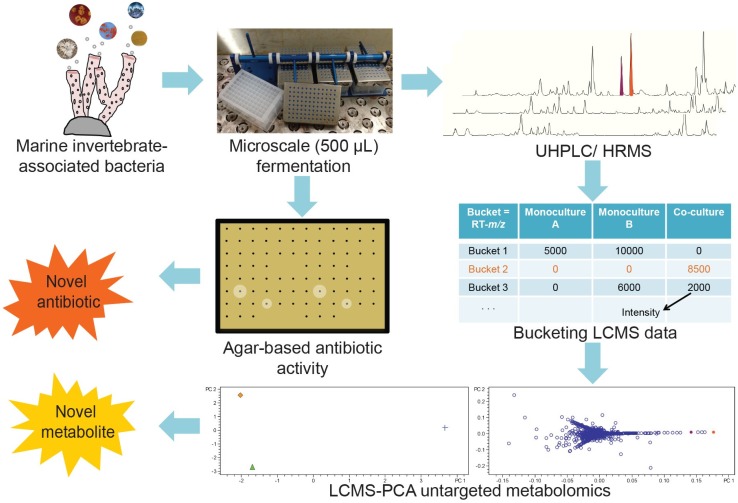
Bacteria isolated from marine invertebrates were subjected to microscale fermentation in 96-deepwell plates. Crude extracts of each well were subjected to both antibiotic assays and LC/MS-based secondary metabolomics. Bucketing of LC/MS spectra yielded tables consisting of RT-*m*/*z* pairs (“buckets”) and intensities for each bucket. LC/MS-PCA was performed to generate a dynamically linked graphical representation of variance between monocultures and co-cultures.

Given that Onaka *et al.* demonstrated induced biosynthesis in co-culture of terrestrial *Streptomyces* spp. co-cultured with mycolic acid-containing bacteria, we evaluated the ability of marine mycolic acid-containing bacteria to induce biosynthesis in marine invertebrate-associated Micromonosporaceae. We sought to first evaluate potential interactions, while also evaluating microscale co-culture platforms to enable broader scale studies. As was recently emphasized in a thorough review by Wolfender and co-workers, advanced analytical methods are necessary to improve detection and identification of metabolites produced in co-cultures *versus* monoculture controls [21]. The co-culture platform described herein evaluates the use of antibiotic activity-based detection in parallel with a more comprehensive LC/MS-based untargeted metabolomics approach coupled with principal component analysis (PCA) (Figure 1). With the ability to analyze large numbers of culture combinations, we sought to miniaturize culture volumes to microscale (500 μL). Importantly, we evaluated co-culture methods both in terms of reproducibility and scalability to larger volumes. To evaluate the methods, we used a set of marine invertebrate-associated Micromonosporaceae that in most cases were not prolific producers of natural products as determined by LC/MS-PCA.

## 2. Results and Discussion

### 2.1. Microscale Fermentation of Monocultures and Co-Cultures

Given the vast number of bacterial combinations, especially from marine invertebrates, we evaluated microscale fermentation as an enabling tool to increase capacity (*i.e.*, numbers of interactions) in a manner that would be scalable for production. Therefore, microscale (500 μL) fermentation of marine invertebrate-associated bacteria in 96-deep-well plates was investigated. We cultured bacteria with a focus on Actinobacteria from marine sponges and ascidians collected in the Florida Keys. On the basis of previously published work by Onaka *et al.*, five mycolic acid-containing bacteria (*Dietzia* sp., *Mycobacterium* sp., *Nocardia* sp., *Rhodococcus* sp., and *Tsukamurella* sp.) were isolated from marine sponges and evaluated as inducers for co-culture. Using microscale fermentation methods similar to those published by Duetz *et al.*, we first evaluated mycolic acid-containing bacteria with *Streptomyces* spp. prior to evaluating Micromonosporaceae [35,36]. There were three major goals for evaluating *Streptomyces* spp. First, we wanted to ensure that we also observed induction by mycolic acid-containing bacteria similar to Onaka’s work. Second, we wanted to confirm that the work could be done in microscale. Third, we wanted to choose the two best mycolic acid-containing bacteria out of the five in order to evaluate a broader range of genera within the Micromonosporaceae. Therefore, a panel of 34 *Streptomyces* spp. were grown in co-culture with each of the five mycolic acid-containing bacteria. On the basis of antibiotic activity produced in co-culture, but not monoculture, *Mycobacterium* sp. (strain WMMA-183) and *Rhodococcus* sp. (strain WMMA-185) produced the most bioactivity. Therefore, the strains of *Mycobacterium* sp. (WMMA-183) and *Rhodococcus* sp. (WMMA-185) were selected for studies to evaluate interspecies interactions within multiple genera of Micromonosporaceae.

**Figure 2:**
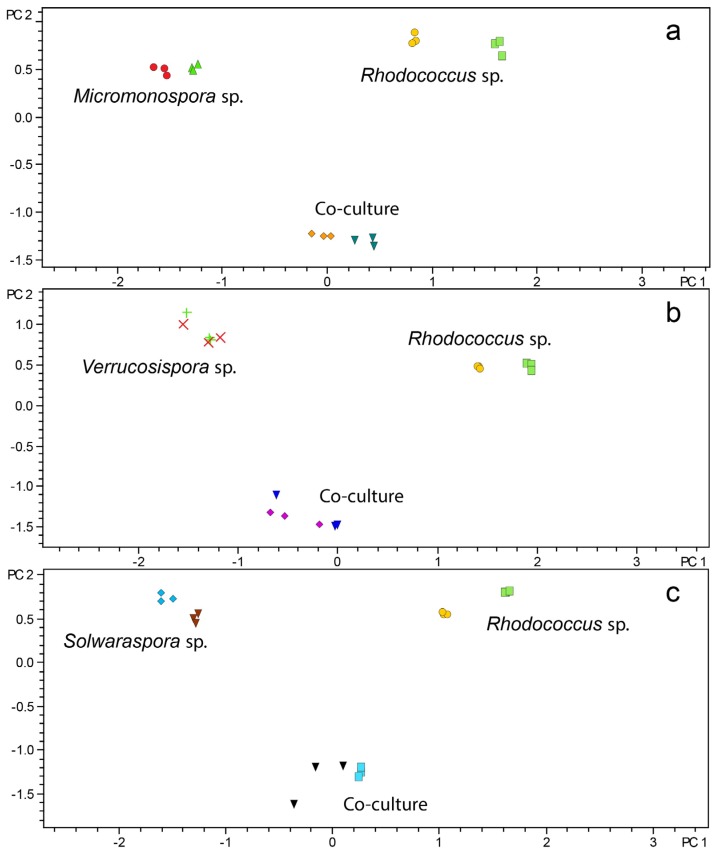
Biological and technical replicates of monocultures and co-cultures *Rhodococcus* sp. cultured with and without (**a**) *Micromonospora* sp. (strain WMMA-1802); (**b**) *Verrucosispora* sp. (strain WMMA-1826); (**c**) *Solwaraspora* sp. (strain WMMA-1845).

While reproducibility was previously documented for *Streptomyces* spp. in microscale fermentation and confirmed by our preliminary experiments, less was known about Micromonosporaceae. To evaluate reproducibility with Micromonosporaceae, a total of six Micromonosporaceae (two *Micromonospora* spp., two *Solwaraspora* spp., and two *Verrucosispora* spp.) were grown in the presence and absence of *Rhodococcus* sp. Monocultures and co-cultures were grown in microscale and crude extracts were subjected to LC/MS-based PCA. Both biological duplicates and technical triplicates were analyzed using PCA scores plots. Spatial clustering of each replicate in the PCA scores plots supported the use of microscale cultivation in combination with metabolomics as a reproducible method for investigating large numbers of bacterial extracts (Figure 2). As shown in Figure 2, the replicate samples grouped together indicating good reproducibility, and the co-cultures did not group with the monocultures indicating that LCMS-PCA was useful for distinguishing the groups and will be discussed in further detail in the section discussing secondary metabolite changes in co-culture.

### 2.2. Evaluation of Induced Antibiotic Production in Micromonosporaceae via Co-Culture with Mycolic Acid-Containing Bacteria

As a starting point to evaluate how mycolic acid-containing bacteria affect secondary metabolite production in Micromonosporaceae, a representative group was evaluated for induction of antibiotics. In total, 65 Micromonosporaceae (47 *Micromonospora* spp., 11 *Verrucosispora* spp., and seven *Solwaraspora* spp.) were selected for analysis via monoculture and co-culture (Table S1, Supporting Information). Antibiotic screening of monoculture and co-cultures was evaluated as a method to detect antibiotic production solely via co-culture. In short, crude extracts were transferred to agar lawns of either Gram-positive bacteria (*B. subtilis*, *MSSA*), Gram-negative bacteria (*E. coli*, *P. aeruginosa*), or yeast (*C. albicans*). Following an 18-hour incubation, instances of growth inhibition halos surrounding spots treated with co-culture extracts, and not corresponding monocultures, were identified as producers of induced antibiotic activity. Antibiotic screening of the 65 Micromonosporaceae in monoculture and co-culture with *Mycobacterium* sp. and *Rhodococcus* sp. resulted in two examples of induced antibiotic production in co-culture, but not monoculture. *Micromonospora* sp. (strain WMMA-1949) inhibited *B. subtilis*, *E. coli*, and Methicillin-sensitive *S. aureus* (MSSA) when co-cultured with either *Mycobacterium* sp. or *Rhodococcus* sp. Similarly, *Micromonospora* sp. (strain WMMB-894) also inhibited *B. subtilis*, *E. coli*, and MSSA, but only when co-cultured with *Rhodococcus* sp. Despite the antibiotic activity observed in co-cultures with *Micromonospora* sp. (strain WMMA-1949), unique metabolites between mono- and co-cultures were not readily identified using LC/MS-based metabolomics. This example highlights the importance of using antibiotic activity as a complementary approach to metabolomics as differences may be overlooked by metabolomics if unique compounds display low ion intensity when analyzed by mass spectrometry. Metabolomics analysis of *Micromonospora* sp. (strain WMMB-894) co-cultured with *Rhodococcus* sp. showed various compounds exclusively produced in co-culture (Figure S9, Supporting Information). Antibiotic activity-based detection provided an advantage in both throughput and therapeutic relevance of induced metabolites. One significant limitation of antibiotic activity-based detection was the potential for overlooking antibiotics produced in co-culture due to initial activity observed in monoculture from an unrelated antibiotic. Furthermore, novel metabolites could be below detection thresholds due to the crude nature of the extracts. In terms of better understanding interspecies interactions among Micromonosporaceae a more comprehensive LC/MS-based metabolomics approach was also used to evaluate metabolite differences in co-culture.

### 2.3. Investigation of Co-Culture by Metabolomics

**Figure 3:**
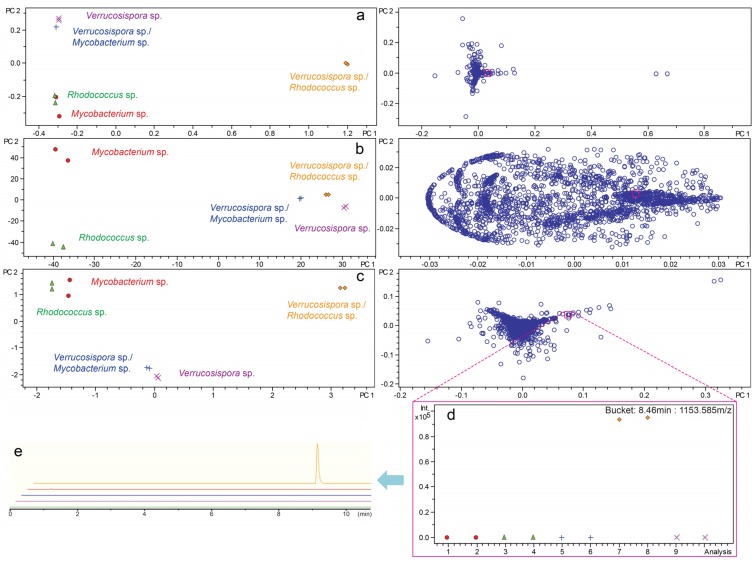
Scores and loadings plots from PCA of monocultures and co-cultures were generated for *Verrucosispora* sp. (strain WMMB-224) in the presence of a *Mycobacterium* sp. (strain WMMA-183) and a *Rhodococcus* sp. (strain WMMA-185). Scaling parameters for LC/MS-PCA were optimized using (**a**) none; (**b**) unit variance; (**c**) Pareto scaling. A bucket statistic was used to display the intensity (*y*-axis) of a unique compound (RT = 8.46 min, *m*/z = 1153.585) identified using Pareto scaling in each sample (*x*-axis) (**d**). An extracted ion chromatogram for each LC/MS chromatogram was used to confirm production of the unique compound in co-culture (**e**).

As a complementary approach to antibiotic activity, co-cultures were analyzed by LCMS-based metabolomics using methods similar to those previously published for strain selection/dereplication by LCMS-PCA [37]. Due to the potential up- and downregulation of metabolite production via co-culture, we wanted to further evaluate the effects of scaling on the outcome of analysis of co-culture by PCA. Importantly, the goal of strain selection by metabolomics was significantly different than understanding changes in metabolites. We evaluated common scaling parameters on PCA for co-cultures of *Verrucosispora* sp. (strain WMMB-224) with *Mycobacterium* sp. and *Rhodococcus* sp. as there were clear differences between the mono- and co-cultures. In the absence of scaling, signals with high intensity dominated the analysis (Figure 3a). Unique, yet low-intensity, metabolites in the loadings plot were overlapped and not readily identified. Using unit variance scaling, variables were divided by the standard deviation, which removed the intensity variable (Figure 3b). Intensity was an important variable to include in our analyses, in particular with regards to up- or downregulation of metabolites. Using Pareto scaling, each variable was divided by the square root of the standard deviation and had been previously used to statistically suppress signals with significant fold-change [37]. Importantly, Pareto scaling provided a balance between the unmodified data and the dimensionless unit variance scaling (Figure 3c). As can be seen in Figure 3d, using Pareto scaling, a specific compound (RT = 8.46 min, *m*/*z* = 1153.585) was readily identified as a uniquely produced metabolite in the *Verrucosispora* sp. and *Rhodococcus* sp. co-culture. The selected compound was further confirmed as present and unique to co-culture by comparison of extracted ion chromatograms for each monoculture and co-culture LC/MS spectrum (Figure 3e). In both the absence of scaling and with unit variance scaling this selected compound was indiscernible likely due to the relatively low ion intensity (0.9 × 10^5^). This example highlights the importance of Pareto scaling for reducing the weight of high-intensity compounds thereby facilitating identification of low intensity ions. These data suggested that Pareto scaling was suitable for evaluating co-cultures.

**Figure 4:**
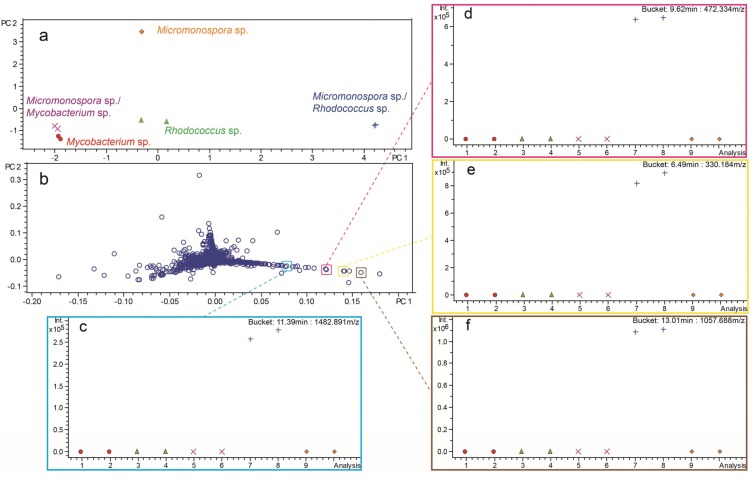
(**a**) PCA scores plot of *Micromonospora* sp. (strain WMMA-1910) in monoculture with *Mycobacterium* sp. (strain WMMA-183) and *Rhodococcus* sp. (strain WMMA-185). Compounds responsible for unique separation of co-cultures from monocultures in scores plot were displayed in the loadings plot (**b**). Example compounds produced exclusively in co-culture were displayed using bucket statistics (**c**–**f**).

Co-cultures producing unique chemistry were identified on the basis of spatial separation from monocultures in PCA scores plots. Compounds influencing the variance observed in the scores plots were identified in loadings plots and subsequently confirmed in corresponding LC/MS chromatograms. As an example, PCA analysis of *Micromonospora* sp. (strain WMMA-1910) and *Rhodococcus* sp. co-culture led to the identification of metabolites produced exclusively in co-culture (Figure 4). Spatial separation of the *Micromonospora* sp. and *Rhodococcus* sp. co-culture to the lower right quadrant of the scores plot indicated a unique chemical profile compared to monocultures and co-culture with *Mycobacterium* sp. (Figure 4a). Individual “buckets” (RT-*m/z* pairs) projecting towards the lower right quadrant of the loadings plot represented compounds unique to co-culture (Figure 4b). Buckets statistics were used to confirm the unique production of four representative compounds in *Micromonospora* sp. and *Rhodococcus* sp. co-culture (Figure 4c–f).

**Figure 5:**
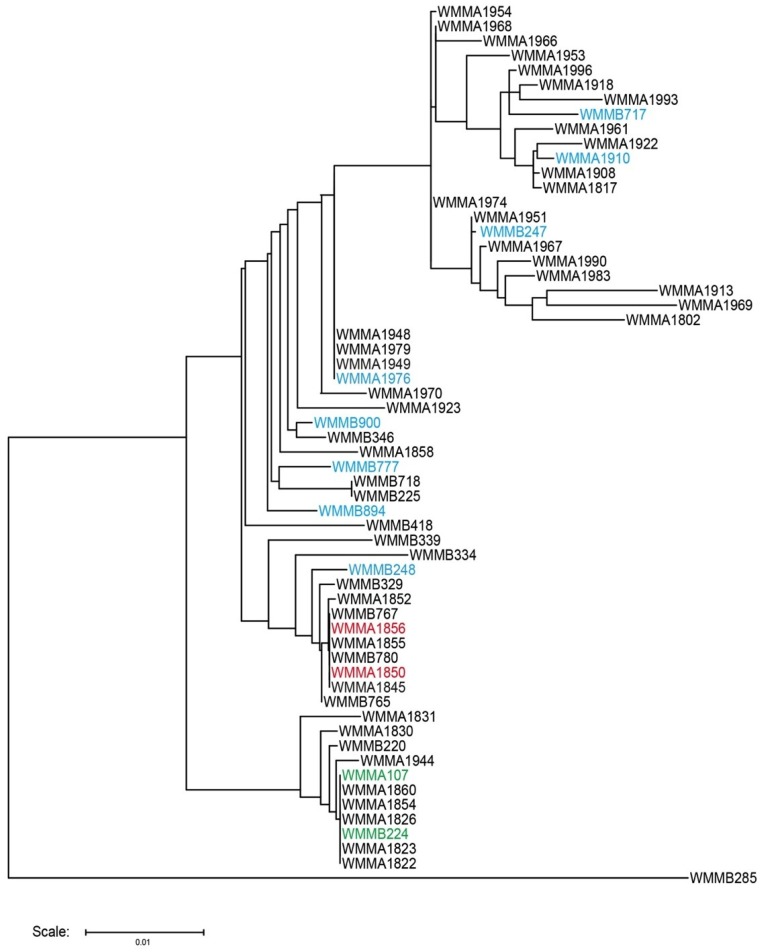
Phylogenetic distribution of *Micromonospora* spp. (**blue**), *Solwaraspora* spp. (**red**), *Verrucosispora* spp. (**green**) producing unique chemistry in co-culture.

PCA, as described for *Micromonospora* sp. (strain WMMA-1910), was performed on 65 Micromonosporaceae and the corresponding 130 co-cultures to evaluate unique metabolite production. A total of 12 Micromonosporaceae (18 co-cultures) exhibited discernable variance in co-culture as compared to monoculture (Table 1). On the basis of PCA analysis, a total of six Micromonosporaceae (12 co-cultures) that produced unique chemistry in co-culture shared similar chemical profiles when co-cultured with *Mycobacterium* sp. and *Rhodococcus* sp. (Figures S1–S6, Supporting Information). In contrast, the remaining six Micromonosporaceae (12 co-cultures) produced different chemistry depending on the bacterium with which it was co-cultured (Figures S7–S10, Supporting Information). In total, 18 co-cultures consisting of eight *Micromonospora* spp., two *Solwaraspora* spp., and two *Verrucosispora* spp. were identified as producers of unique chemistry. A phylogenetic tree was generated to display the distribution of the 12 Micromonosporaceae producing unique chemistry via co-culture (Figure 5). Analysis of the phylogenetic diversity indicated the presence of interspecies interactions across all three genera investigated.

**Table 1:** Co-cultures producing unique bioactivity (□) and/or secondary metabolites (■).

Strain	Organism	*Mycobacterium* sp.	*Rhodococcus* sp.
WMMA-1850	*Solwaraspora* sp.		■
WMMA-1856	*Solwaraspora* sp.	■	■
WMMA-1910	*Micromonospora* sp.		■
WMMA-1949	*Micromonospora* sp.	□	□
WMMA-1976	*Micromonospora* sp.	■	■
WMMA-107	*Verrucosispora* sp.		■
WMMB-224	*Verrucosispora* sp.		■
WMMB-247	*Micromonospora* sp.	■	■
WMMB-248	*Micromonospora* sp.		■
WMMB-717	*Micromonospora* sp.	■	■
WMMB-777	*Micromonospora* sp.	■	■
WMMB-894	*Micromonospora* sp.		□ ■
WMMB-900	*Micromonospora* sp.	■	■

**Figure 6:**
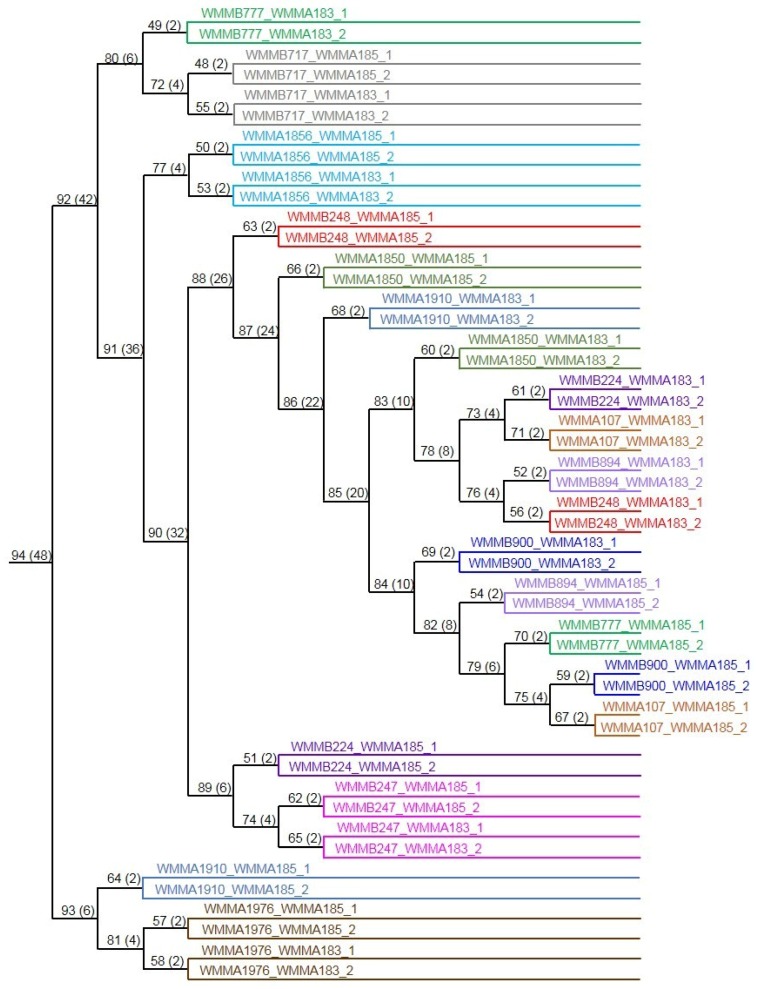
Hierarchical clustering of LC/MS chromatograms of co-cultures producing unique metabolites.

While identification of 18 co-cultures producing unique chemistry was promising, there was potential that the outcome was not strain specific meaning that the new metabolites in all co-cultures were the same. To understand the relationships among the co-cultures, similarity in chemical profiles among the 24 co-cultures were evaluated using LC/MS-based Hierarchical clustering. Using Hierarchical cluster analysis, co-cultures producing similar chemical profiles were clustered, as was observed for co-cultures of *Micromonospora* sp. (strain WMMB-717) with both *Mycobacterium* sp. and *Rhodococcus* sp. (Figure 6). Few instances of clustering between co-cultures of different Micromonosporaceae indicated metabolites produced in co-culture were strain specific (Figure 6).

Secondary metabolites exclusively produced in co-culture were further evaluated for novelty. Continuing with the example *Verrucosispora* sp. (strain WMMB-224) co-culture (Figure 3), a total of 29 compounds were identified as being exclusively produced in co-culture with *Rhodococcus* sp. High-resolution mass data were used to query a natural product database (Antibase) to determine novelty. In total, 27 metabolites were determined to be putative novel compounds based on Antibase searches against high resolution masses and simulated molecular formulas using Bruker DataAnalysis 4.2. Larger scale (100 mL) fermentations were grown to evaluate scalability of the metabolites produced in co-culture of *Verrucosispora* sp. (strain WMMB-224) and *Rhodococcus* sp. Of the 27 putative novel metabolites identified via microscale fermentation, 24 were reproduced in larger scale. These results demonstrate our ability to identify putative novel metabolites from microscale fermentation and analysis of hundreds of co-culture combinations and subsequently pursue target molecules in larger scale making our approach amenable to identification of novel molecules with therapeutic applications.

## 3. Experimental Section

### 3.1. Strain Collection and Selection

Strains used in this study were isolated from sponge or ascidian specimens in the Florida Keys, FL USA. Voucher specimens of both ascidians and sponges used in this study were housed at the University of Wisconsin-Madison. For cultivation, a sample of invertebrate (1 cm^3^) was rinsed with sterile seawater, macerated with a sterile pestle in a microcentrifuge tube, and diluted with sterile seawater. Each dilution was plated separately on three media: ISP2, R2A, and M4. Each medium was supplemented with 50 μg/mL cycloheximide and 25 μg/mL nalidixic acid and incubated at 28 °C for at least 28 days. Nearly full length 16S rDNA sequencing was performed using previously described protocols [38]. A complete list of strains used and their associated sequence identity were tabulated (Table S1, Supporting Information.). Phylogenetic tree was created using Tree Builder software on RDP website with default settings [39]. Due to incomplete sequences, strains WMMA-1955, WMMA-1959, WMMA-1972, WMMA-1980, WMMA-1982, and WMMA-1992 were not included in phylogenetic tree. It should also be noted that none of these strains were identified as being relevant to the current studies.

### 3.2. Microscale Cultivation

Seed cultures were initially grown in 10 mL seed cultures (25 × 150 mm tubes) in ASW-A media (20 g soluble starch, 10 g glucose, 5 g peptone, 5 g yeast extract, 5 g CaCO_3_ per liter of artificial seawater). Detoxified polypropylene square 96-deepwell microplates (Enzyscreen, The Netherlands) were autoclaved separately from the ASW-A media. Following sterilization, 500 μL ASW-A was added to each well. Co-culture wells were inoculated with 15 μL of either *Streptomyces* sp. or Micromonosporaceae and 5 μL of mycolic acid-containing bacteria. Monocultures controls were inoculated with identical volumes as co-culture wells. Culture plates were covered using low-evaporation sandwich covers (Enzyscreen, The Netherlands). Culture plates were mounted into orbital shakers using CR1700 cover clamps (Enzyscreen, The Netherlands). Culture plates were incubated at 30 °C for fourteen days and shaken at 300 rpm for optimal oxygen transfer rates for reproducibility at larger scale [35,36].

### 3.3. Microscale Extraction

Following fourteen-day growth, 500 μL of MeOH was added to each well of the culture plate. Cultures were mixed with the MeOH and allowed to soak for 60 min. Culture plates were placed in a SpeedVac for 90 min on medium heat to pellet cells and remove MeOH. The crude extract supernatant (500 µL), was divided equally into two shallow 96-well plates for either downstream chemical analysis or antibiotic activity screening.

### 3.4. Antibiotic Activity Screening

Supernatant from culture plates were tested against *B. subtilis*, *E. coli*, Methicillin-sensitive *S. aureus* (MSSA), *C. albicans*, and *P. aeruginosa* using an agar-based antibiotic assay. Methods were modified from a previously reported yeast halo assay [40]. Seed cultures of each screening strain were grown overnight in 10 mL in LB media (25 × 150 mm tubes). To prepare uniform lawns for screening, 4 mL of overnight culture was inoculated into 200 mL of Cation-adjusted Mueller-Hinton Agar (CAMHA) (1.5 g soluble starch, 17.5 g casein, 3 g beef extract, 15 g agar, 12.5 mg Mg^2+^, 25 mg Ca^2+^, in 1 L di-H_2_O) maintained at 50–55 °C. For each screening plate, 30 mL of inoculated agar was poured into OmniTray (Thermo Scientific, Waltham, MA, USA) and cooled for 30 min. From 96-well format, 5 μL of extract was spotted onto the agar. Screening plates were incubated at 37 °C overnight and subsequently visually observed for zones of inhibition.

### 3.5. Sample Processing of Microscale Cultures for UHPLC/HRESI-TOF-MS Analysis

Half of the total crude extract was split into a designated plate for chemical analysis. A solution of 10:1 H_2_O:MeOH (250 μL) was added to each well containing dried extracts. Solubilized extracts were transferred to 1 dram vials and diluted to a final volume of 1 mL using 10:1 H_2_O:MeOH. Using a Gilson GX-271 liquid handling system, 900 μL was subjected to automated solid phase extraction (SPE). Extracts were loaded onto pre-conditioned (1 mL MeOH followed by 1 mL H_2_O) EVOLUTE ABN SPE cartridges (25 mg absorbent mass, 1 mL reservoir volume; Biotage, Charlotte, NC, USA). Samples were subsequently washed using H_2_O (1 mL) to remove media components, and eluted with MeOH (500 μL) directly into an LC/MS-certified vial.

### 3.6. Sample Analysis of Microscale Cultures via UHPLC/HRESI-TOF-MS

LC/MS data were acquired using a Bruker MaXis ESI-Q-TOF mass spectrometer (Bruker, Billerica, MA, USA) coupled with a Waters Acquity UPLC system (Waters, Milford, MA, USA) operated by Bruker Hystar software. A gradient comprised of MeOH and H_2_O (containing 0.1% formic acid) was used on an RP C-18 column (Phenomenex Kinetex 2.6 μm, 2.1 mm × 100 mm; Phenomenex, Torrance, CA, USA) at a flow rate of 0.3 mL/min. The method consisted of a linear gradient from MeOH/H_2_O (10%/90%) to MeOH/H_2_O (97%/3%) in 12 min, then held for two minutes at MeOH/H_2_O (97%/3%). Full scan mass spectra (*m*/*z* 150–1550) were measured in positive ESI mode. The mass spectrometer was operated using the following parameters: capillary, 4.5 kV; nebulizer pressure, 1.2 bar; dry gas flow, 8.0 L/min; dry gas temperature, 205 °C; scan rate, 2 Hz. Tune mix (ESI-L low concentration; Agilent, Santa Clara, CA) was introduced through a divert valve at the end of each chromatographic run for automated internal calibration. Bruker Data Analysis 4.2 was used for analysis of chromatograms.

### 3.7. Bucketing and PCA of LC/MS Data

Bucketing LC/MS data and PCA was performed using Bruker Profile Analysis 2.1. LC/MS bucketing was performed using the following parameters: data selection and processing, Find Molecular Features; spectrum type, line; spectrum polarity, positive; spectral range, 2–14 min and *m*/*z* = 150–1500; advanced bucketing, 20 seconds and 20 mDa window; normalization, Sum of bucket values in analysis. PCA was performed on bucket tables using a Pareto scaling algorithm.

## 4. Conclusions

Our study utilizes an improved co-culture approach to investigate the prevalence of interspecies interactions within Micromonosporaceae from the marine invertebrate niche. Both bioassay-guided LC/MS-guided detection were used to identify a total of 20 co-cultures producing either unique bioactivity and/or secondary metabolites in co-culture (Table 1). Owing to the differences in chemical profile between 12 co-culture combinations depending on which inducer strain was used, our results indicated an advantage to co-culturing Micromonosporaceae with more than one mycolic acid-containing bacterium. The microscale co-culture approach described herein can facilitate an increase in co-culture combinations. Importantly, our culture-dependent method used to study Micromonosporaceae was superior to genome-dependent methods (*i.e.*, metagenomics) in part due to the limited genomic information available for Micromonosporaceae to date. Far fewer Micromonosporaceae genomes have been characterized as compared to other biosynthetically rich bacteria. As of August 2015, compared to the 180 *Streptomyces* spp. genomes reported in NCBI, eight *Micromonospora* spp., one *Verrucosispora* sp., and 0 *Solwaraspora* sp. were reported. In addition to novel antibiotics and secondary metabolites, our metabolomics investigations may be used to identify novel genes based on correlation to unique chemistry. In particular, biosynthetic genes, or genes responsible for biosynthetic regulation, would be valuable outcomes of future co-culture experiments.

## Supplementary Information

Complete list of bacteria used in study is provided. LCMS-PCA Scores and Loadings Plots for all Micromonosporaceae producing unique chemistry via co-culture are included. This material is available free of charge via MDPI website.
