# Supplementary material for: Investigation of Interspecies Interactions within Marine Micromonosporaceae Using an Improved Co-Culture Approach
Source: Mar Drugs. 2015 Sep 24;13(10):6082–98. doi: 10.3390/md13106082 (PMC4626680; doi:10.3390/md13106082)
Supplement: Supplementary file 1 [file marinedrugs-13-06082-s001.docx]

**Supplementary Information**

**Table of Contents Page**

**Table S1.** List of bacteria used in study S1

**Figure S1.** LCMS-PCA Scores and Loadings Plots of WMMB-717 S3

**Figure S2.** LCMS-PCA Scores and Loadings Plots of WMMB-247 S4

**Figure S3.** LCMS-PCA Scores and Loadings Plots of WMMA-1856 S4

**Figure S4.** LCMS-PCA Scores and Loadings Plots of WMMB-777 S5

**Figure S5.** LCMS-PCA Scores and Loadings Plots of WMMB-900 S5

**Figure S6.** LCMS-PCA Scores and Loadings Plots of WMMA-1976 S5

**Figure S7.** LCMS-PCA Scores and Loadings Plots of WMMA-1850 S6

**Figure S8.** LCMS-PCA Scores and Loadings Plots of WMMB-248 S6

**Figure S9.** LCMS-PCA Scores and Loadings Plots of WMMB-894 S6

**Figure S10.** LCMS-PCA Scores and Loadings Plots of WMMA-107 S7

**Table S1.** List of 65 Micromonosporaceae and 2 mycolic acid-containing bacteria used in study.

| **Strain #** | **Taxonomic Information** |
| --- | --- |
| WMMA-1802 | *Micromonospora* sp. |
| WMMA-1817 | *Micromonospora* sp. |
| WMMA-1822 | *Verrucosispora* sp. |
| WMMA-1823 | *Verrucosispora* sp. |
| WMMA-1826 | *Verrucosispora* sp. |
| WMMA-1830 | *Verrucosispora* sp. |
| WMMA-1831 | *Verrucosispora* sp. |
| WMMA-1845 | *Solwaraspora* sp. |
| WMMA-1850 | *Solwaraspora* sp. |
| WMMA-1852 | *Solwaraspora* sp. |
| WMMA-1854 | *Verrucosispora* sp. |
| WMMA-1855 | *Solwaraspora* sp. |
| WMMA-1856 | *Solwaraspora* sp. |
| WMMA-1858 | *Micromonospora* sp. |
| WMMA-1860 | *Verrucosispora* sp. |
| WMMA-1908 | *Micromonospora* sp. |
| WMMA-1910 | *Micromonospora* sp. |
| WMMA-1913 | *Micromonospora* sp. |
| WMMA-1918 | *Micromonospora* sp. |
| WMMA-1922 | *Micromonospora* sp. |
| WMMA-1923 | *Micromonospora* sp. |
| WMMA-1944 | *Verrucosispora* sp. |
| WMMA-1948 | *Micromonospora* sp. |
| WMMA-1949 | *Micromonospora* sp. |

**Table S1.** *Cont.*

| WMMA-107 | *Verrucosispora* sp. |
| --- | --- |
| WMMB-220 | *Verrucosispora* sp. |
| WMMB-224 | *Verrucosispora* sp. |
| WMMB-225 | *Micromonospora* sp. |
| WMMB-247 | *Micromonospora* sp. |
| WMMB-248 | *Micromonospora* sp. |
| WMMB-329 | *Solwaraspora* sp. |
| WMMB-334 | *Solwaraspora* sp. |
| WMMB-339 | *Micromonospora* sp. |
| WMMB-346 | *Micromonospora* sp. |
| WMMB-418 | *Micromonospora* sp. |
| WMMB-717 | *Micromonospora* sp. |
| WMMB-718 | *Micromonospora* sp. |
| WMMB-765 | *Micromonospora* sp. |
| WMMB-767 | *Micromonospora* sp. |
| WMMB-777 | *Micromonospora* sp. |
| WMMB-780 | *Micromonospora* sp. |
| WMMB-894 | *Micromonospora* sp. |
| WMMB-900 | *Micromonospora* sp. |
| WMMA-1951 | *Micromonospora* sp. |
| WMMA-1953 | *Micromonospora* sp. |
| WMMA-1954 | *Micromonospora* sp. |
| WMMA-1955 | *Micromonospora* sp. |
| WMMA-1959 | *Micromonospora* sp. |
| WMMA-1961 | *Micromonospora* sp. |
| WMMA-1966 | *Micromonospora* sp. |
| WMMA-1967 | *Micromonospora* sp. |
| WMMA-1968 | *Micromonospora* sp. |
| WMMA-1969 | *Micromonospora* sp. |
| WMMA-1970 | *Micromonospora* sp. |
| WMMA-1972 | *Micromonospora* sp. |
| WMMA-1974 | *Micromonospora* sp. |
| WMMA-1976 | *Micromonospora* sp. |
| WMMA-1979 | *Micromonospora* sp. |
| WMMA-1980 | *Micromonospora* sp. |
| WMMA-1982 | *Micromonospora* sp. |
| WMMA-1983 | *Micromonospora* sp. |
| WMMA-1990 | *Micromonospora* sp. |
| WMMA-1992 | *Micromonospora* sp. |
| WMMA-1993 | *Micromonospora* sp. |
| WMMA-1996 | *Micromonospora* sp. |
| WMMA-183 | *Mycobacterium* sp. |
| WMMA-185 | *Rhodococcus* sp. |


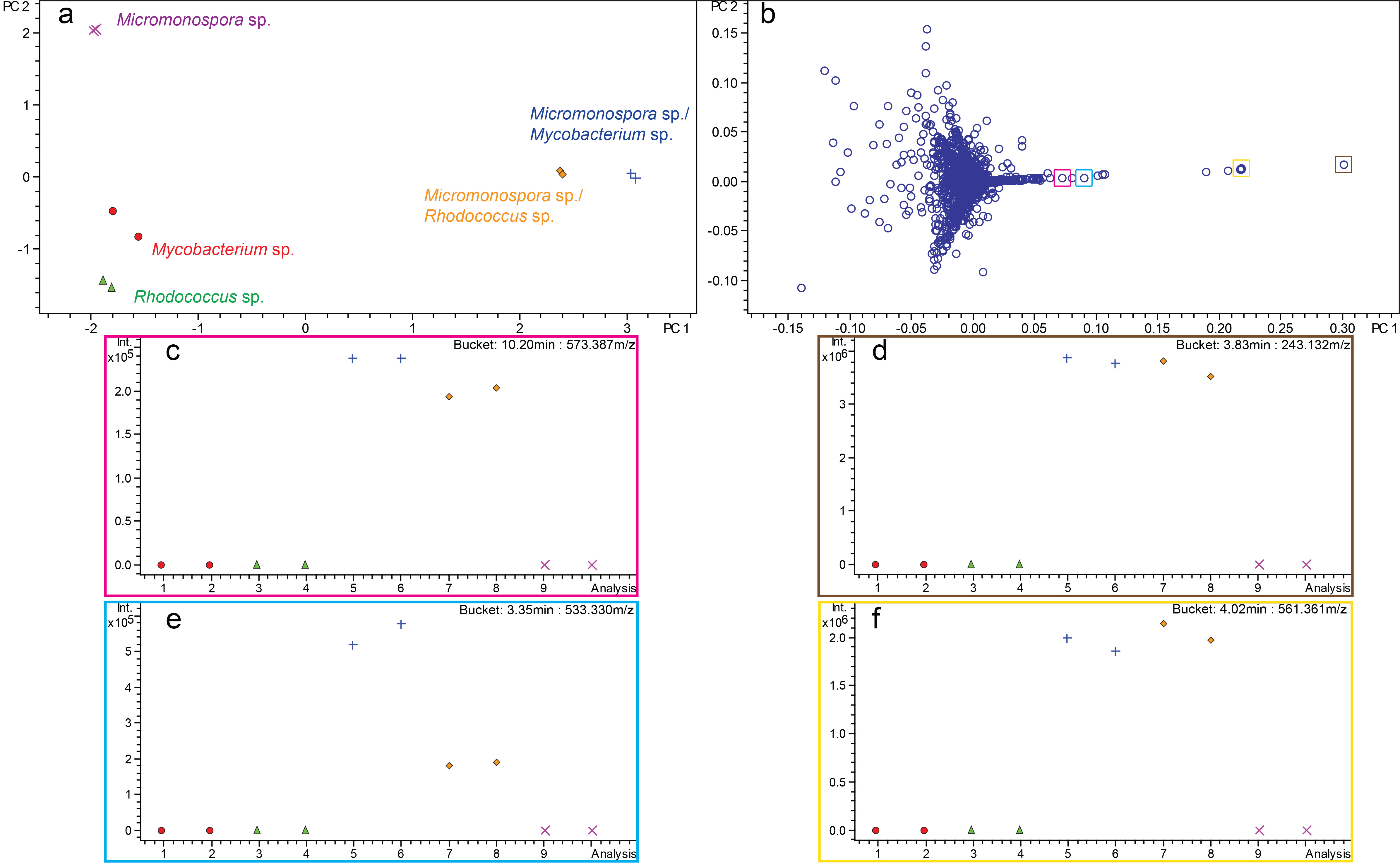


**Figure S1.** (**a**) PCA scores pot of *Micromonospora* sp. (strain WMMB-717) in monoculture and co-culture with *Mycobacterium* sp. (WMMA-183) and *Rhodococcus* sp. (WMMA-185); (**b**) Loadings plot displaying compounds responsible for unique separation of co-cultures from monocultures in scores plot; (**c**–**f**) Bucket statistics of example compounds produced exclusively in co-culture.


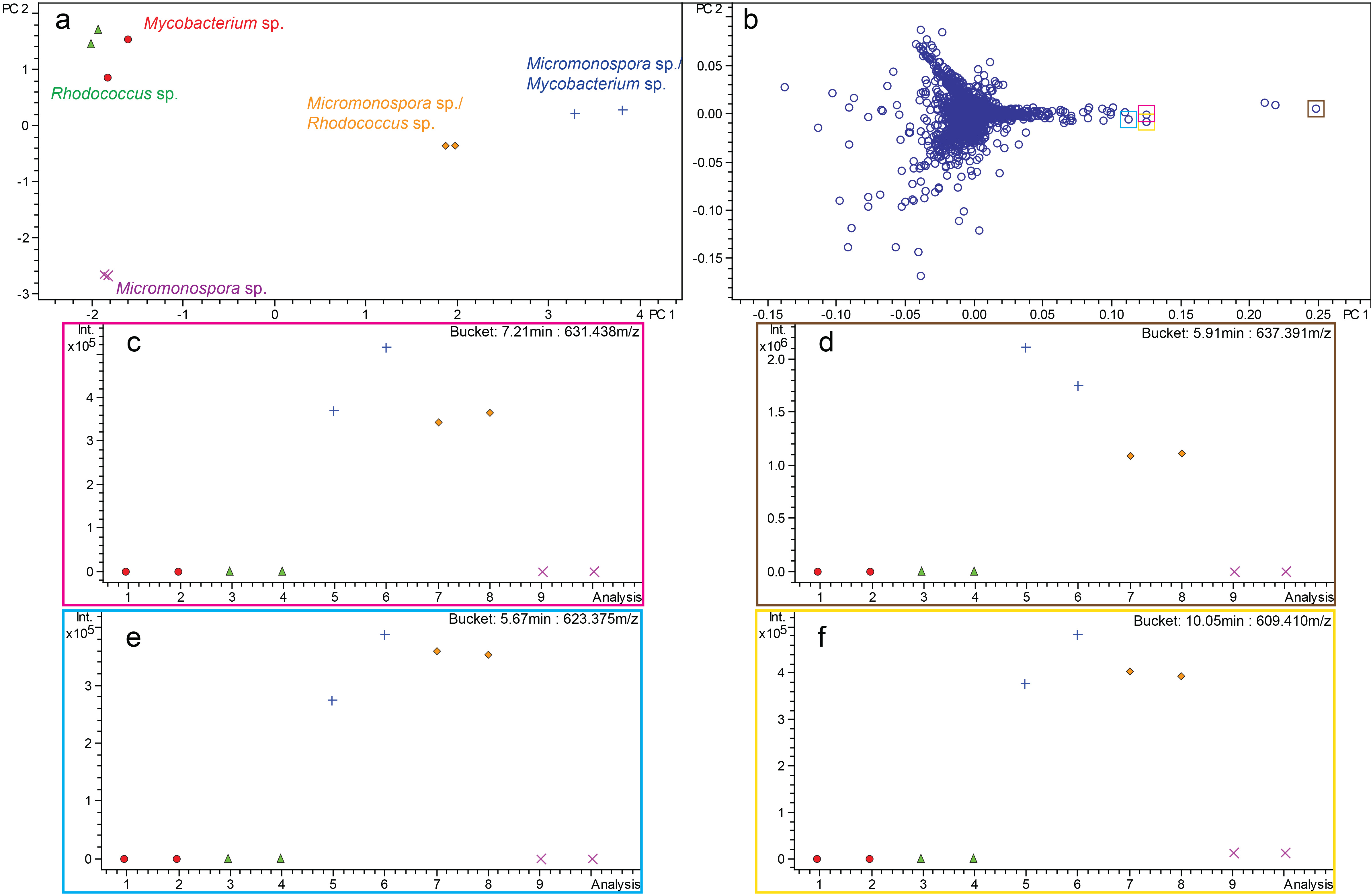


**Figure S2.** (**a**) PCA scores pot of *Micromonospora* sp. (strain WMMB-247) in monoculture and co-culture with *Mycobacterium* sp. (WMMA-183) and *Rhodococcus* sp. (WMMA-185); (**b**) Loadings plot displaying compounds responsible for unique separation of co-cultures from monocultures in scores plot; (**c**–**f**) Bucket statistics of example compounds produced exclusively in co-culture.


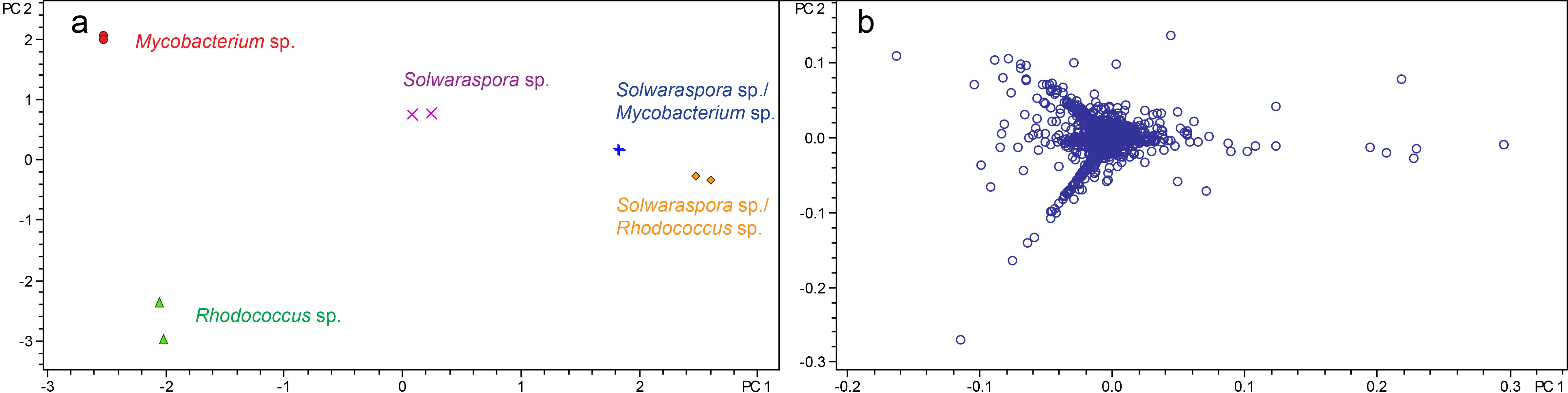


**Figure S3.** (**a**) PCA scores pot of *Solwaraspora* sp. (strain WMMA-1856) in monoculture and co-culture with *Mycobacterium* sp. (WMMA-183) and *Rhodococcus* sp. (WMMA-185); (**b**) Loadings plot displaying compounds responsible for unique separation of co-cultures from monocultures in scores plot.


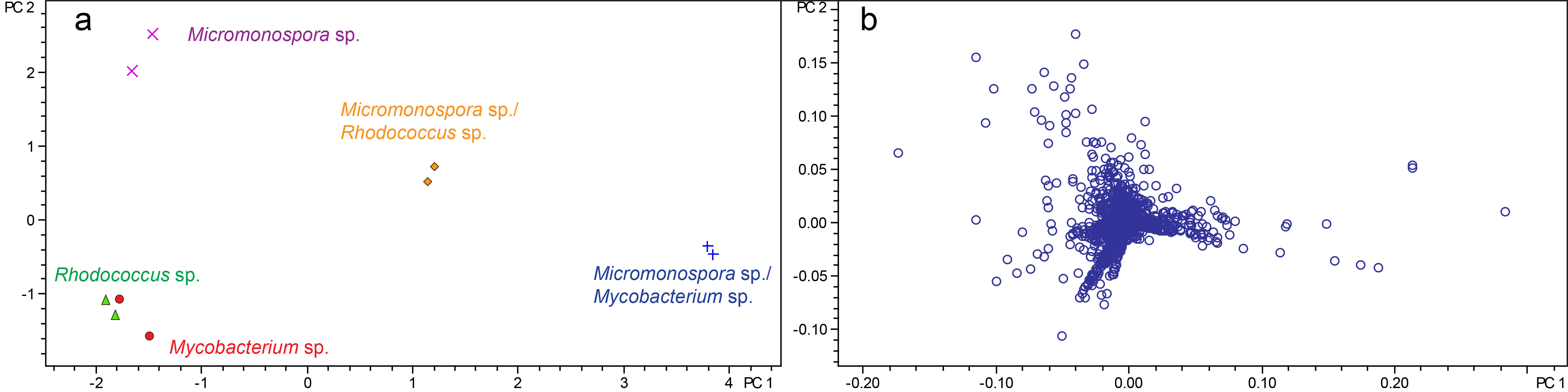


**Figure S4.** (**a**) PCA scores pot of *Micromonospora* sp. (strain WMMB-777) in monoculture and co-culture with *Mycobacterium* sp. (WMMA-183) and *Rhodococcus* sp. (WMMA-185); (**b**) Loadings plot displaying compounds responsible for unique separation of co-cultures from monocultures in scores plot.


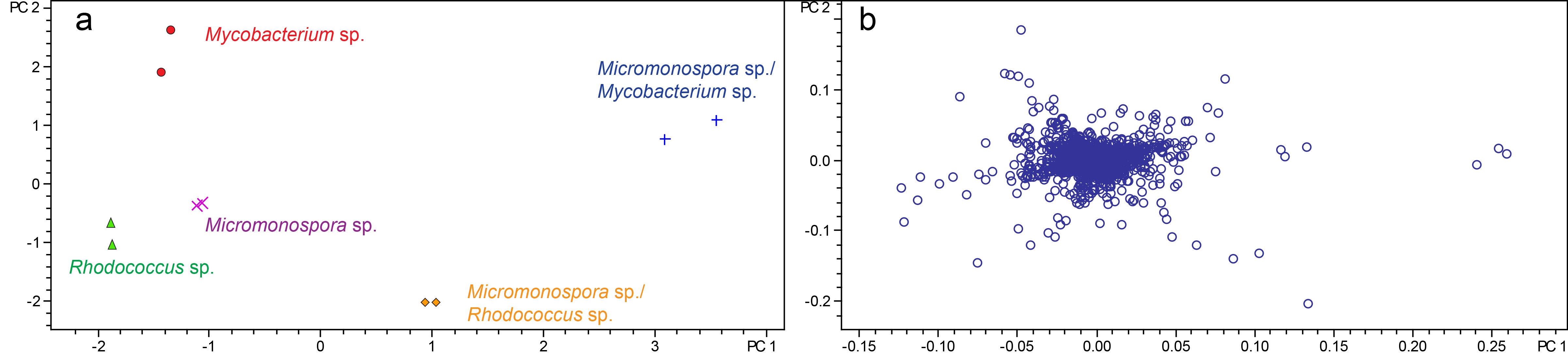


**Figure S5.** (**a**) PCA scores pot of *Micromonospora* sp. (strain WMMB-900) in monoculture and co-culture with *Mycobacterium* sp. (WMMA-183) and *Rhodococcus* sp. (WMMA-185); (**b**) Loadings plot displaying compounds responsible for unique separation of co-cultures from monocultures in scores plot.


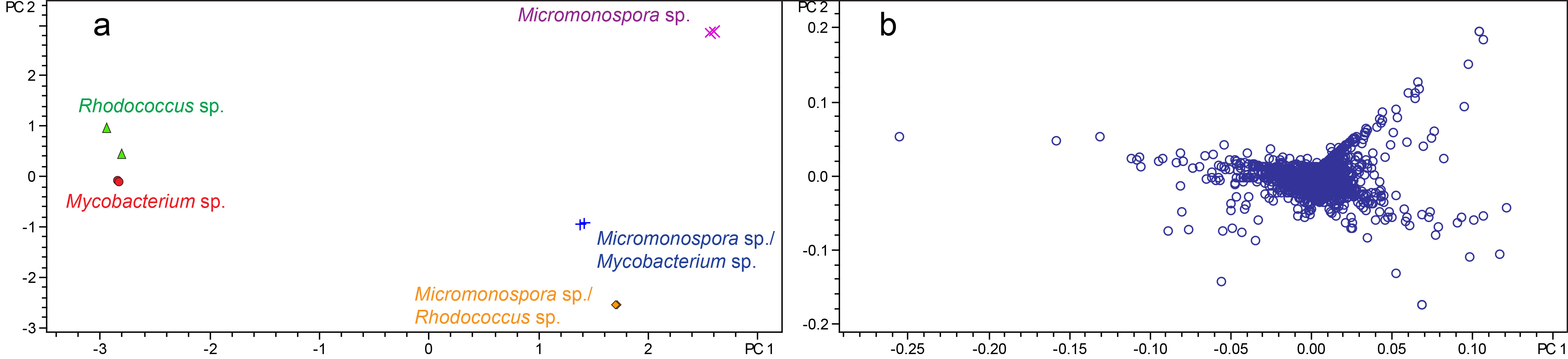


**Figure S6.** (**a**) PCA scores pot of *Micromonospora* sp. (strain WMMA-1976) in monoculture and co-culture with *Mycobacterium* sp. (WMMA-183) and *Rhodococcus* sp. (WMMA-185); (**b**) Loadings plot displaying compounds responsible for unique separation of co-cultures from monocultures in scores plot.


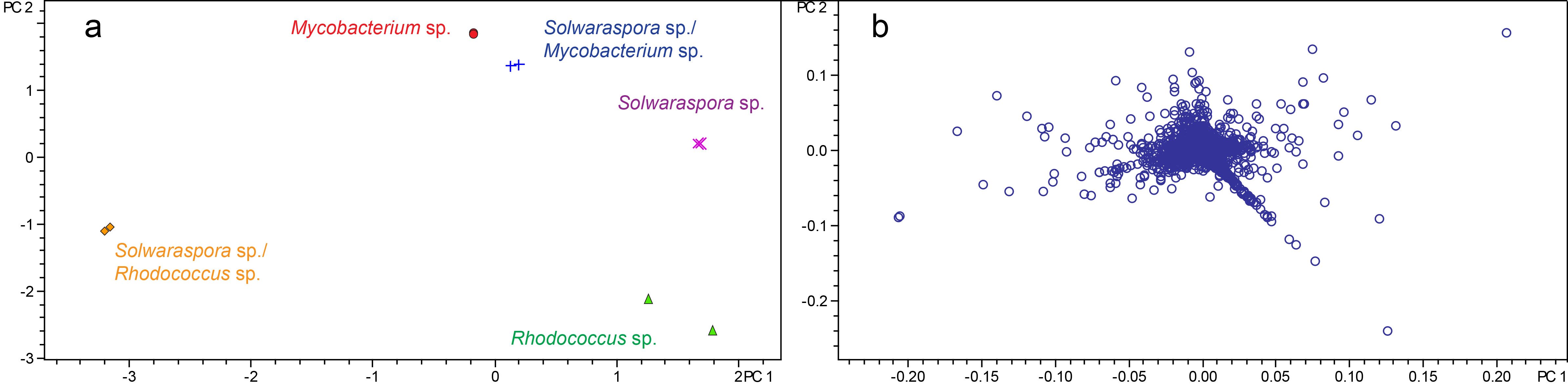


**Figure S7.** (**a**) PCA scores pot of *Solwaraspora* sp. (strain WMMA-1850) in monoculture and co-culture with *Mycobacterium* sp. (WMMA-183) and *Rhodococcus* sp. (WMMA-185); (**b**) Loadings plot displaying compounds responsible for unique separation of co-cultures from monocultures in scores plot.


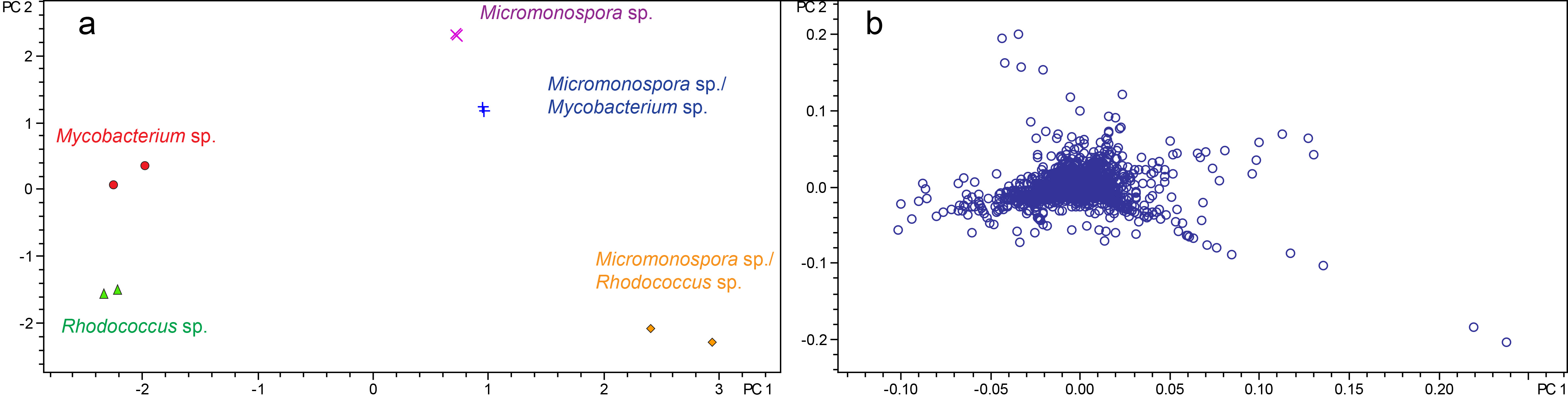


**Figure S8.** (**a**) PCA scores pot of *Micromonospora* sp. (strain WMMB-248) in monoculture and co-culture with *Mycobacterium* sp. (WMMA-183) and *Rhodococcus* sp. (WMMA-185); (**b**) Loadings plot displaying compounds responsible for unique separation of co-cultures from monocultures in scores plot.


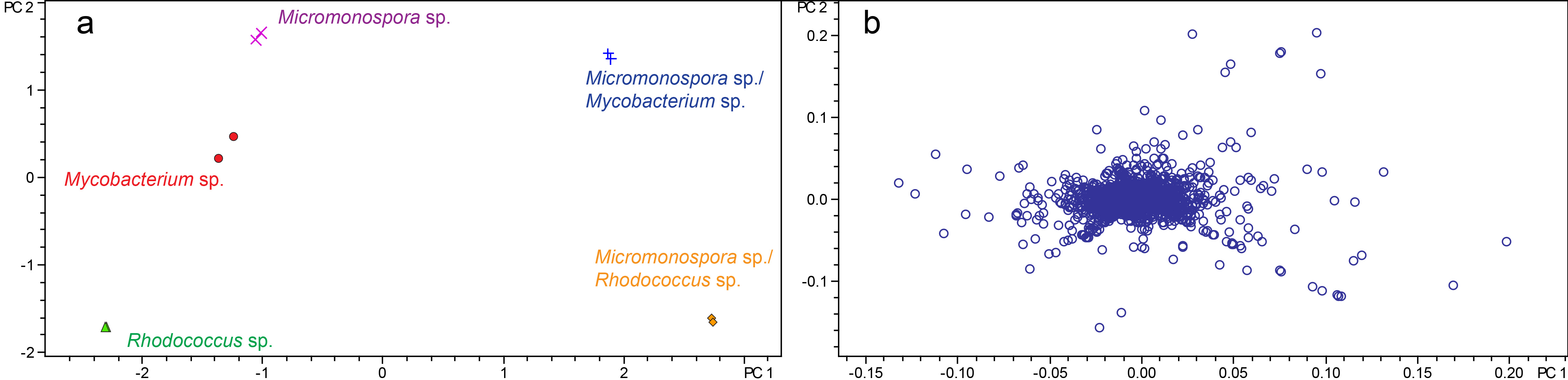


**Figure S9.** (**a**) PCA scores pot of *Micromonospora* sp. (strain WMMB-894) in monoculture and co-culture with *Mycobacterium* sp. (WMMA-183) and *Rhodococcus* sp. (WMMA-185); (**b**) Loadings plot displaying compounds responsible for unique separation of co-cultures from monocultures in scores plot.


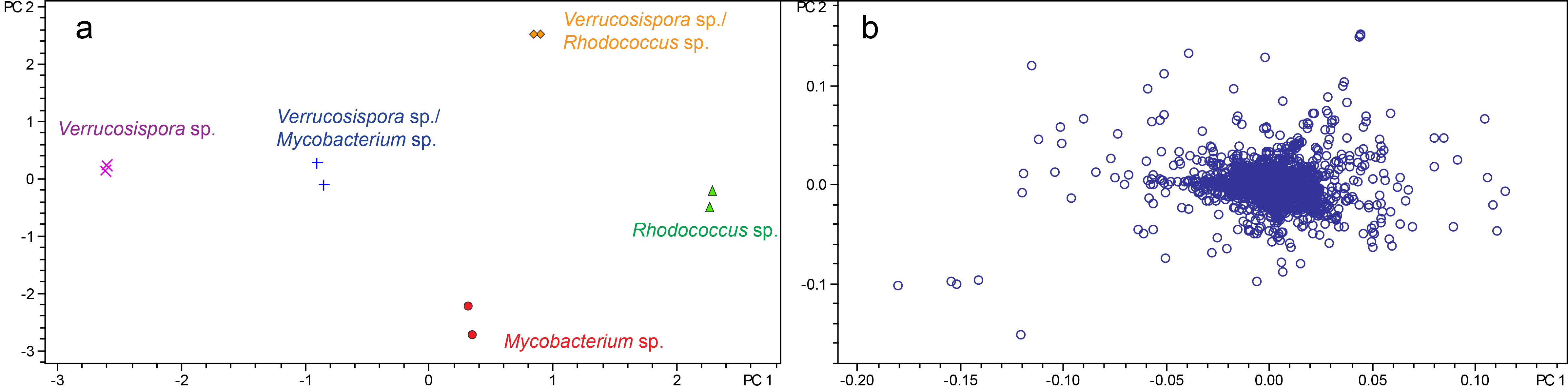


**Figure S10.** (**a**) PCA scores pot of *Verrucosispora* sp. (strain WMMA-107) in monoculture and co-culture with *Mycobacterium* sp. (WMMA-183) and *Rhodococcus* sp. (WMMA-185); (**b**) Loadings plot displaying compounds responsible for unique separation of co-cultures from monocultures in scores plot.

© 2015 by the authors; licensee MDPI, Basel, Switzerland. This article is an open access article distributed under the terms and conditions of the Creative Commons Attribution license (http://creativecommons.org/licenses/by/4.0/).
